# Molecular and Functional Signatures Associated with CAR T Cell Exhaustion and Impaired Clinical Response in Patients with B Cell Malignancies

**DOI:** 10.3390/cells11071140

**Published:** 2022-03-28

**Authors:** Katia Beider, Orit Itzhaki, Jacob Schachter, Ania Hava Grushchenko-Polaq, Valeria Voevoda-Dimenshtein, Evgenia Rosenberg, Olga Ostrovsky, Olivia Devillers, Ronnie Shapira Frommer, Li-at Zeltzer, Amos Toren, Elad Jacoby, Avichai Shimoni, Abraham Avigdor, Arnon Nagler, Michal J. Besser

**Affiliations:** 1 Division of Hematology and Bone Marrow Transplantation, Chaim Sheba Medical Center, Tel Aviv University, Tel Aviv 6997801, Israel; katiabeider@gmail.com (K.B.); aniahava.grushchenkopolaq@sheba.health.gov.il (A.H.G.-P.); lerush86@gmail.com (V.V.-D.); jeniarosen@gmail.com (E.R.); olga.ostrovsky@sheba.health.gov.il (O.O.); oliviadevillers1@gmail.com (O.D.); avichai.shimoni@sheba.health.gov.il (A.S.); abraham.avigdor@sheba.health.gov.il (A.A.); 2 Ella Lemelbaum Institute for Immuno Oncology, Chaim Sheba Medical Center, Tel Aviv 6997801, Israel; orit.itzhaki@sheba.health.gov.il (O.I.); jacob.schachter@sheba.health.gov.il (J.S.); ronnie.shapira@sheba.health.gov.il (R.S.F.); liat.zeltzer@sheba.health.gov.il (L.-a.Z.); 3 Center for Pediatric Cell Therapy, Chaim Sheba Medical Center, Tel Aviv University, Tel Aviv 6997801, Israel; amos.toren@sheba.health.gov.il (A.T.); elad.jacoby@sheba.health.gov.il (E.J.); 4 Department of Clinical Microbiology and Immunology, Sackler School of Medicine, Tel Aviv University, Tel Aviv 6997801, Israel

**Keywords:** CAR T, B cell malignancies, exhaustion, resistance, molecular signature

## Abstract

Despite the high rates of complete remission following chimeric antigen receptor (CAR) T cell therapy, its full capacity is currently limited by the generation of dysfunctional CAR T cells. Senescent or exhausted CAR T cells possess poor targeting and effector functions, as well as impaired cell proliferation and persistence in vivo. Strategies to detect, prevent or reverse T cell exhaustion are therefore required in order to enhance the effectiveness of CAR T immunotherapy. Here we report that CD19 CAR T cells from non-responding patients with B cell malignancies show enrichment of CD8^+^ cells with exhausted/senescent phenotype and display a distinct transcriptional signature with dysregulation of genes associated with terminal exhaustion. Furthermore, CAR T cells from non-responding patients exhibit reduced proliferative capacity and decreased IL-2 production in vitro, indicating functional impairment. Overall, our work reveals potential mediators of resistance, paving the way to studies that will enhance the efficacy and durability of CAR T therapy in B cell malignancies.

## 1. Introduction

Chimeric antigen receptor (CAR) T cell therapy has shown promising results in patients with B cell malignancies. The high rates of remission induced by the CAR T cells in relapsed/refractory CD19-expressing B cell malignancies have led to FDA approval of several anti-CD19 CAR T cell products for the treatment of both diffuse large B cell lymphoma (DLBCL) and B cell acute lymphoblastic leukemia (ALL) [1,2,3]. However, despite the initial response, less than 50% of patients treated with CAR T cells experience long-term disease control [4]. Therefore, further efforts are needed to improve the outcomes of these patients. Expansion, persistence and tumor cytotoxicity are among the main characteristics of CAR T cells that shape treatment efficacy. However, the fundamental mechanisms underlying CAR T unresponsiveness in patients with B cell malignancies remain poorly understood.

T cell exhaustion has been implicated as an important factor limiting the efficacy of CAR T cells against cancer [5,6]. T cell dysfunction comprises several differentiation programs of functional unresponsiveness, including T cell exhaustion, anergy and senescence [7]. Exhaustion refers to a state of T cell dysfunction characterized by a decrease in effector function and increased expression of inhibitory receptors, usually induced by chronic antigen stimulation during unresolved chronic infection or cancer [8]. Exhausted T cells progressively lose their ability to produce interleukin-2 (IL-2), proliferative capacity and capability to mediate ex vivo killing. Exhausted T cells exhibit a distinct phenotype including increased expression of inhibitory receptors, (e.g., programmed cell death -1 (PD-1), cytotoxic T-lymphocyte-associated protein 4 (CTLA-4), T-cell immunoglobulin and ITIM domain (TIGIT), lymphocyte activating 3 (LAG-3), T-cell immunoglobulin mucin-3 (TIM3)) and impaired ability to release tumor necrosis factor (TNF) and interferon-γ (IFN γ) [8,9]. T cell exhaustion was initially described in a mouse model of lymphocytic choroid meningitis virus, or LCMV infection [10]. In the setting of chronic infection, exhausted “progenitor” T cells with memory-like phenotype retain polyfunctionality, persist during chronic antigen stimulation, and differentiate into “terminally” exhausted T cells [8]. Subsequently, first studies of dysfunctional T cells in tumors suggested that they share features of T cell exhaustion [11,12]. Later work has suggested that cancer-associated T cell dysfunction is a unique state that is distinct from T cell exhaustion [13,14]. However, cellular and molecular characteristics of CAR T cell dysfunction and diminished clinical responses in patients with B cell malignancies are not fully elucidated.

The current work aims to identify phenotypic, transcriptional and cellular mediators of resistance to CAR T immune therapy. Here we reveal a cellular and molecular signature as a predictive indicator of clinical response. Enrichment of differentiated exhausted CD8^+^ T cells was identified as the main feature of CAR T cells in patients with B cell malignancies failing to respond to CAR T cell therapy. Interestingly, elevated levels of differentiated CD8^+^ T cells were already observed in the peripheral blood of patients prior to CAR T therapy. Furthermore, the transcriptomic state of CAR T cells with exhausted phenotype displayed distinct cytokine expression profiles with increased expression of exhaustion and terminal differentiation markers. Finally, CAR T cells with an exhausted phenotype demonstrate functional impairment, manifested by low IL-2 production and decreased ex vivo proliferative capacity.

## 2. Methods

### 2.1. Patients and CAR-T Protocol

A total of 42 patients with relapsed or refractory B cell malignancies were included in this study. These patients were participants in a phase 1b/2 study of locally produced CD19 CAR T cells (NCT02772198) conducted at the Sheba Medical Center, Tel-Hashomer, Israel. The study was approved by the Institutional Review Board and the Israeli Ministry of Health in accordance with the Declaration of Helsinki. Briefly, patients underwent a single leukapheresis procedure, peripheral blood mononuclear cells were isolated, activated and transduced with a gamma retrovirus encoding for a CD19 CAR (based on an FMC63 derived ScFv, a CD28 costimulatory domain and CD3-zeta signaling domain). Lymphodepletion included fludarabine 25 mg/m^2^ for 3 days (days -4 to -2) and cyclophosphamide 900 mg/m^2^ for 1 day (day -2), followed by infusion (day 0) of 1–1.5 × 10^6^ CAR+ transduced cells per kilogram recipient. Aliquots from the CAR T cell products pre-infusion, patient peripheral blood (PB) samples pre-lymphodepletion and PB samples pre-CAR T cell administration were analyzed.

### 2.2. Peripheral Blood Mononuclear Cell Separation and Cryopreservation

Peripheral blood mononuclear cells (PBMCs) were isolated from the peripheral blood of patients prior to lymphodepletion by density gradient with Ficoll-Hypaque (Pharmacia Biotech, Piscataway, NJ, USA). CD3^+^ untouched lymphocytes were separated from the peripheral blood of patients before lymphodepletion by RosetteSep^TM^ Human T Cell Enrichment Cocktail (StemCell Technologies, Vancouver, Canada) using negative selection. Briefly, 4 mL of whole blood was mixed with 200 µL RosetteSep™ Cocktail and centrifuged over a density gradient medium (Ficoll-Hypaque, Pharmacia Biotech, Piscataway, NJ, USA). CD3^+^ enriched population at the interface between the plasma and the density gradient medium was collected.

### 2.3. RNA Extraction

Purified CD3^+^ and CAR T cells (300,000 cells) were used for RNA extraction using NucleoSpin RNA Plus XS kit (Machery-Nagel, Duren, Germany) according to the manufacturer’s specifications; on-column DNase digestion was also performed to remove genomic DNA. Extracted RNA was quantified using a NanoDrop 2000 spectrophotometer (Thermo Scientific Inc. Wilmington, NC, USA).

### 2.4. Gene Expression Analysis

Gene expression analysis was performed at NanoString Technologies (Seattle, WA, USA). RNA was used as input into the nCounter^®^ CAR T Characterization Gene Expression Panel (NanoString Technologies), a 770-plex gene expression panel that profiles the human immune response. Raw data were imported from the MAX digital analyzer into nSolver software v3.0 (NanoString Technologies). Standard quality control checks assessing imaging, binding density, positive control linearity and limit of detection were performed. By utilizing internal positive controls in the panels, raw gene expression data were first normalized. The messenger RNA (mRNA) data were further normalized by the geometric mean of a set of stably expressed reference genes. The mRNA expressed below background were filtered from the analysis using cutoffs of mean plus two standard deviations of negative controls.

### 2.5. RT-PCR Analysis

To generate cDNA, 1 μg total RNA was reverse transcribed using the qScript cDNA Synthesis Kit (Quanta Biosciences, Beverly, MA, USA) according to the manufacturer’s instructions. Real-time quantitative PCR (RT-qPCR) was performed in a final volume of 20 μL, containing 50 ng of total RNA-derived cDNAs, forward and reverse primers (300 nM) and PerfeCta SYBR Green FastMix (Quanta Biosciences, Beverly, MA, USA), using the StepOnePlus Real-Time PCR system (Applied Biosystems, San Francisco, CA, USA). Changes in expression levels were normalized to control β2-microglobulin using the ΔΔCT method of relative quantification using the StepOne Software v2.2. Experiments were performed in triplicates for each sample. The sequences of primers are presented in Supplementary Table S1.

### 2.6. Phenotype and Activation Status

Aliquots from manufactured CAR T cell batches or PBMCs from patient blood were analyzed by flow cytometry stained with fluorescent-labeled antibodies against those listed in Supplementary Table S3. FITC conjugated recombinant human CD19 protein (Sino Biologicals Inc, Wayne, PA, USA) was used to determine CAR expression. Data was acquired using Navios Beckman Coulter instrument and analyzed with Kaluza Analysis Software version 2.1.

### 2.7. Cell Lines and Cell Culture

CD19-positive Raji lymphoma cells and CD19-negative THP-1 monocytic leukemia cells were purchased from American Type Culture Collection and maintained in log-phase growth in RPMI1640 medium (Biological Industries, Kibbutz Beit-Haemek, Israel) supplemented with 10% heat-inactivated fetal calf serum (FCS), 1 mM L-glutamine, 100 U/mL penicillin and 0.01 mg/mL streptomycin (Biological Industries, Kibbutz Beit-Haemek, Israel) in a humidified atmosphere of 5% CO_2_ at 37 °C.

### 2.8. CAR T Ex Vivo Proliferation

Raji target tumor cells were labeled with carboxyfluorescein succinimidyl ester (CFSE) (Thermo Fisher Scientific, Waltham, MA USA) according to the manufacturer’s instructions. CAR T cells (5 × 10^4^) were cocultured with CFSE-labeled Raji cells at 1:1, 1:0.5 and 1:0.25 effector to target ratio [E: T] for 5 days. The presence of IL-2 in the supernatant was quantified by enzyme-linked immunosorbent assay (Invitrogen, Waltham, MA USA) according to the manufacturer’s instructions. The number of proliferated CAR T cells was determined by flow cytometry, with the addition of 123 count eBeads™ Counting Beads (Thermo Fisher Scientific, Waltham, MA USA), using Beckman Coulter instrument and analyzed using Kaluza Software.

### 2.9. Cytotoxic Activity

Raji and THP-1 target cells were labeled with CFSE according to the manufacturer’s instructions. Approximately 5 × 10^4^ Raji or THP-1 target cells were co-cultured with 10 × 10^4^, 5 × 10^4^ or 2.5 × 10^4^ expanded CD19 CAR T cells for 4 h in a 37 °C incubator with 5% CO2. After 4 h, plates were centrifuged at 500× *g* for 5 min, supernatants were removed for cytokine measurements, cells were stained with propidium iodide (PI) (Sigma Aldrich, Rehovot, Israel) at final concentration of 1 μg/mL and analyzed by flow cytometry on a Navios Beckman Coulter instrument and Kaluza Software. Viable PI-negative CFSE-positive target cells were enumerated.

### 2.10. Statistical Analysis

Significance of variation between groups was evaluated using a non-parametric two-tailed Student’s *t*-test. The differences between proportions were tested using two-sided Fisher’s exact test.

### 2.11. Data Availability

The data generated in this study are available within the article and its Supplementary Data Files.

## 3. Results

### 3.1. Patients’ Characteristics and Clinical Response

Between January 2019 and January 2021, patients with relapsed/refractory (r/r) B-cell malignancies were enrolled in the trial. Of the 42 patients included in this study, 32 patients had non-Hodgkin lymphoma (NHL), including DLBCL (*n* = 24), mantle cell lymphoma (*n* = 3) and follicular lymphoma (*n* = 5); 5 patients had acute lymphoblastic leukemia (ALL); and 5 patients had chronic lymphocytic leukemia (CLL) in Richter transformation. Clinical response was evaluated 1 to 2 months after CAR-T cell administration. Patient demographic and clinical characteristics, toxicity and clinical outcome are presented in Table 1. Of the 42 evaluated patients, 28 (67%) achieved complete or partial responses (CR *n* = 24, PR *n* = 4), while in 14 (33%) patients the disease progressed (PD).

### 3.2. CAR T Cell Phenotype Characterization

**Figure 1:**
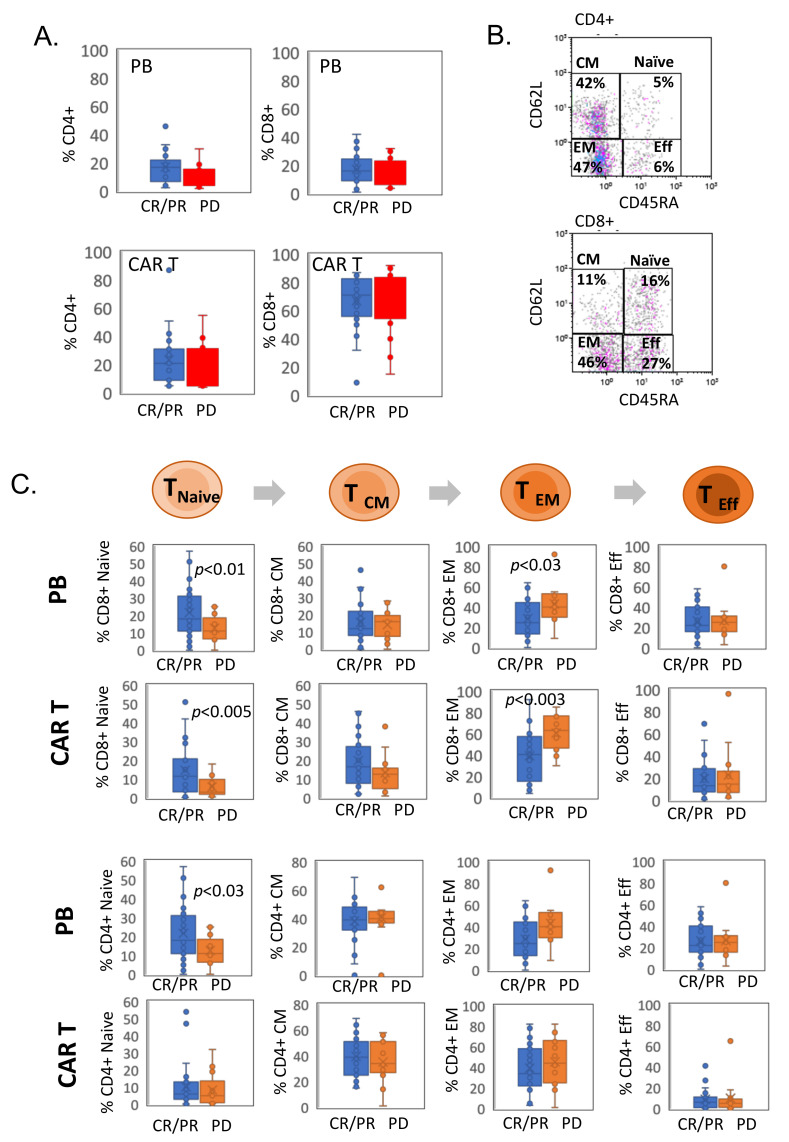
CAR T cell phenotype characterization. (**A**) Frequency of CD4^+^ and CD8^+^ cells in the peripheral blood (PB) mononuclear cells at the time of leukapheresis and in manufactured pre-infused CAR T cell products from patients in each response group (CR/PR, *n* = 28; PD, *n* = 14). (**B**) Representative flow cytometry dot plots examining the proportions of naive (CD45RA^+^CD62L^+^), central memory (CM; CD45RA^-^CD62L^+^), effector memory (EM; CD45RA^-^CD62L^-^) and effector (Eff; CD45RA^+^CD62L^-^) subsets in CD8^+^ and CD4^+^ lymphocytes taken from CAR T cell product of non-responding patient. The cells were pre-gated on CD4^+^ and CD8^+^ populations. (**C**) The percentages of naive, CM, EM and Eff CD8^+^ and CD4^+^ T cells in the PB at the time of leukapheresis and in manufactured pre-infused CAR T cell products from patients in each response group (CR/PR, *n* = 28; PD, *n* = 14).

The percentage of circulating CD4^+^ and CD8^+^ cells in the PB of responding and non-responding patients at baseline (pre-CAR administration) was similar, CD4^+^ 18.3% ± 11% in CR/PR vs. 11.6% ± 8% in PD; CD8^+^ 15.4% ± 10% in CR/PR vs. 15.7% ± 8% in PD. Similarly, manufactured CAR T products were enriched in T cells, with similar CD4^+^ and CD8^+^ frequencies in responders and non-responders, CD4^+^ 21.7% ± 13% in CR/PR vs. 18.2% ± 14.5% in PD; CD8^+^ 68.7% ± 14% in CR/PR vs. 75.2% ± 14% in PD (Figure 1A). CD45RA and CD62L were used to distinguish between several maturation states of CD8^+^ and CD4^+^ T cells (Figure 1B).

We found a significantly increased percentage of differentiated effector memory (EM) within the cytotoxic T cell population, defined as CD62L^-^CD45RA^-^CD8^+^ cells (38.2% ± 19% in CR/PR vs. 64.9% ± 19% in PD, *p* < 0.005), and a reduced percentage of naive CD62L ^+^ CD45RA^+^ CD8^+^ cells (15.5% ± 12% in CR/PR vs. 5.4% ± 5% in PD *p* < 0.004) in CAR T products of non-responding patients relative to responders. Of note, an increased frequency of EM CD8+ cells was already detected in the pre-apheresis PB of non-responding patients (24.1% ± 14% in CR/PR vs. 40.2% ± 15% in PD, *p* < 0.004) (Figure 1C). Differentiated EM T cells are a heterogeneous cell population with enhanced cytolytic activity and limited proliferative capacity, which is enriched with antigen-experienced, exhausted and senescent T cells [15].

### 3.3. CD8+ CAR T Cells Exhibit Features of Exhaustion

**Figure 2:**
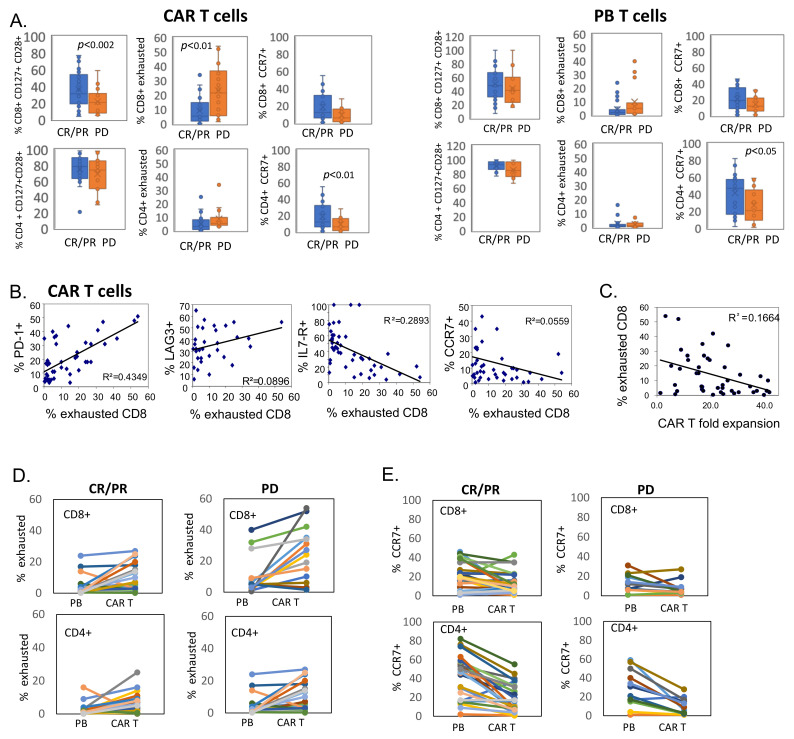
CD8^+^ T cells in peripheral blood and pre-infused CAR T cell products from non-responding patients display phenotypical features of exhaustion. Pre-infused CAR T cell products (**A**) and PB samples at the time of leukapheresis were analyzed by multiparameter flow cytometry. Frequency of CD28^+^CD127^+^, exhausted population (CD28^-^CD57^+^CD39^+^) and CCR7 in CD8^+^ and CD4^+^ T cells was established in each group (CR/PR, *n* = 28; PD, *n* = 14). (**B**) Spearman’s rho correlation (two-tailed) between the expression of PD1, LAG3, CD127 (IL-7R) and CCR7 on CD8^+^ T cells and the frequency of exhausted (CD28^-^CD57^+^CD39^+^) CD8+ cells in pre-infused CAR T cell products. (**C**) Spearman’s rho correlation (two-tailed) between the frequency of exhausted (CD28^-^CD57^+^CD39^+^) CD8+ cells in pre-infused CAR T cell products and CAR T product fold expansion during production. (**D**) Frequency of exhausted CD8^+^ and CD4^+^ cells in paired samples of PB and manufactured CAR T cells from CR/PR and PD patients. (**E**) CCR7 expression in CD8^+^ and CD4^+^ cells in paired samples of PB and manufactured CAR T cells from CR/PR and PD patients.

After showing an increase in EM phenotype in the PB and CAR T cell product of patients not responding to CAR T cells, we next assessed T cells for differentiation and exhaustion markers. Thus, further characterization revealed a significantly lower percentage of CD8^+^ CAR T cells expressing CD127 and CD28 in non-responding patients (36.5% ± 21% in CR/PR vs. 19.9% ± 12% in PD, *p* < 0.002). In addition, a higher percentage of CD8^+^ CAR T cells from non-responding patients expressed CD57 and CD39, cell-surface markers of cellular exhaustion. The cumulative frequency of CD8^+^ CAR T cells with high CD57, high CD39 and low CD28 expression (defined as exhausted population) was significantly higher in the manufactured CAR T products of non-responding patients compared to products of responding patients (9.1% ± 9% in CR/PR vs. 23.1% ± 17.7% in PD, *p* < 0.01). Consistently, higher CCR7 expression was detected on CD4^+^ and CD8^+^ CAR T cells from responding patients in comparison to non-responders, suggesting that a less differentiated phenotype together with increased trafficking of CAR T to lymphoid tissue relates with improved clinical responses. In contrast to the CD8^+^ fraction, CD4^+^ CAR T cells demonstrated a high frequency of CD127^+^CD28^+^ cells and a low frequency of CD57^hi^CD39^hi^CD28^low^ cells regardless of the outcome of the patient (Figure 2A, Supplementary Figure S1). Our data suggest that CD4^+^ are less prone to exhaustion compared to CD8^+^ counterparts, suggesting better persistence of CD4 CAR T cells following antigen exposure.

The presence of immunosuppressive cells, such as regulatory T (Treg) cells, may diminish CAR T responses [16]. A low percentage of CD25^+^CD127^-^ CD4^+^ Treg cells was detected in CAR T products, with no correlation to clinical responses (Supplementary Figure S2). In addition, expression levels of chemokine receptors CXCR3, CXCR4 and CCR5 were similar in responding and non-responding patients (Supplementary Figure S2).

Notably, the CD57^hi^CD39^hi^CD28^low^ ‘exhausted’ phenotype in CD8^+^ CAR T cells corresponded with a higher expression of checkpoint markers such as PD-1 and LAG-3 (Figure 2B), known to be expressed on terminally exhausted T cells [15]. In line with these findings, an increased frequency of exhausted CD8^+^ CAR T cells inversely correlated with CD127 and CCR7 expression in manufactured CD8^+^ CAR T cells (Figure 2B).

T cells with differentiated and exhausted phenotypes have limited proliferative capacity. Therefore, we evaluated the expansion of CAR T cells throughout the clinical production and calculated fold expansion on day 10 compared with day 2 (transduction day). Importantly, the fold expansion on day 10 inversely correlated with increased frequency of CD57^hi^CD39^hi^CD28^low^ CD8^+^ CAR T cells in the infusion product (R^2^ = 0.1664) (Figure 2C). These data suggest that acquisition of differentiated phenotypes with enhanced exhausted/senescent markers may be associated with reduced proliferative capacity during CAR T production.

To evaluate the origin of the CD57^hi^CD39^hi^CD28^low^ phenotype in the manufactured CD8+ CAR T cells, we compared the frequency of this population in paired samples of PB and CAR T cell products from responding and non-responding patients. As presented in Figure 2D, the frequency of CD57^hi^CD39^hi^CD28^low^ CD4^+^ and CD8^+^ in responding patients only mildly increased upon CAR T manufacturing process. In contrast, CD8^+^ CAR T cells of non-responders profoundly up-regulated exhaustion markers upon ex vivo transduction and expansion. Furthermore, increased frequency of exhausted CD8^+^ T cells was detected in baseline PB of three patients with PD and remained high in the manufactured CD8^+^ CAR T cells (Figure 2D). These results suggest that an exhausted T cell population may pre-exist in the PB of patients with lymphatic malignancies, while the manufacturing process, ex vivo activation and expansion further contribute to the acquisition of the exhaustion phenotype that is associated with diminished clinical responses to CAR T therapy.

### 3.4. Transcriptional Profile of CAR T Products

**Figure 3:**
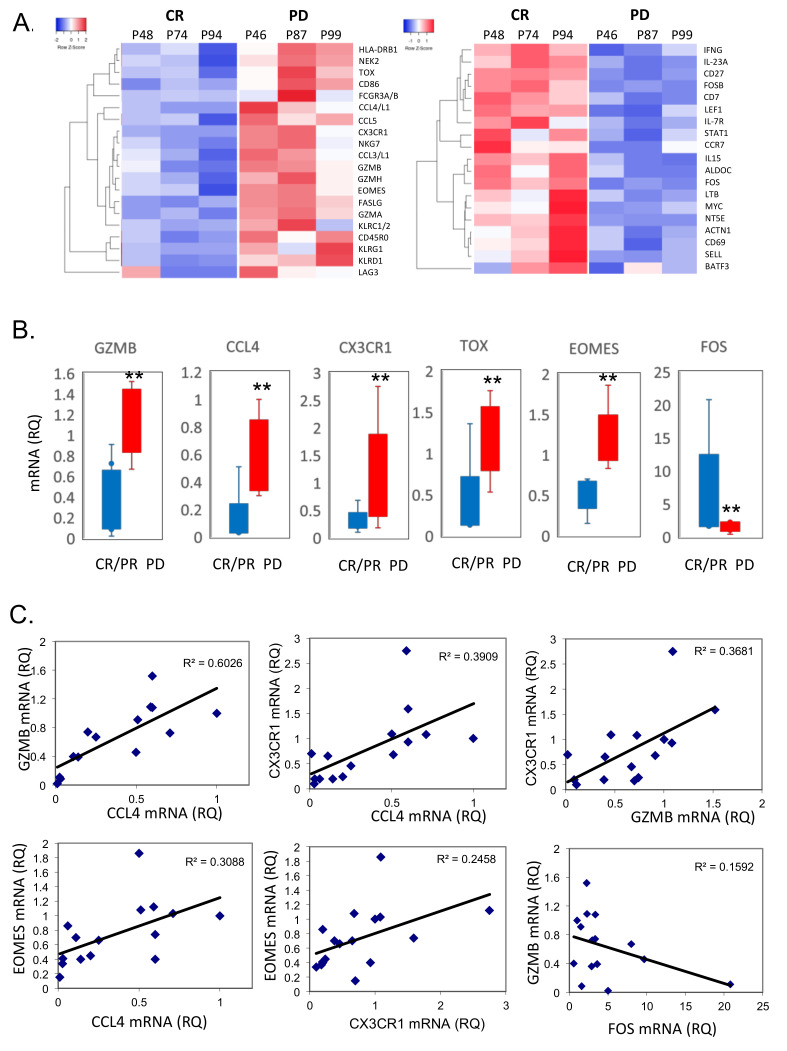
Transcriptional profiles of CAR T cellular products reveal T cell-intrinsic quality attributes associated with clinical response. (**A**) Genes differentially expressed in CAR T cells from patients with complete response (CR) and a low frequency of phenotypically exhausted (CD28^-^CD57^+^CD39^+^) CD8^+^ cells (*n* = 3) and CAR T cells from patients with progressive disease (PD) and a high frequency of exhausted CD8^+^ T cells (*n* = 3). RNA was isolated, and gene expression analysis was performed using the nCounter^®^ CAR-T Characterization Gene Expression Panel (Nanostring). Heat maps show log2-fold gene expression changes (false discovery rate values, 0.01) in CR vs. PD subsets. Each column depicts an individual sample, and each row represents an individual gene, shaded to indicate normalized expression. (**B**) Real-time PCR analysis of relative mRNA expression of *GZMB*, *CCL4*, *CX3CR1*, *TOX*, *EOMES* and *FOS* in CAR-T cells from patients with CR (*n* = 9) and PD (*n* = 6), ** *p* < 0.001. (**C**) Spearman’s rho correlation (two-tailed) between the mRNA expression levels of *GZMB* and *CCL4*, *CX3CR1* and *CCL4*, *CXC3CR1* and *GZMB*, *EOMES* and *CCL4*, *EOMES* and *CX3CR1*, and *GZMB* and *FOS* in CAR T cells (*n* = 15).

We have shown that non-responding patients have a higher proportion of CD57^hi^CD39^hi^CD28^low^ cells in the CAR T cells product, correlating with higher PD-1 and lower CD127 expression. To study whether the transcriptomic profile of CAR T cell products differs between responding and non-responding patients, we used the nCounter^®^ CAR-T Characterization Gene Expression Panel (Nanostring). Products from three complete responders (diagnosed with DLBCL, DLBCL and ALL) and three non-responding patients (diagnosed with DLBCL, ALL and CLL) were analyzed and differentially expressed genes were selected based on a fold change (FC) of greater than 2 between CR and PD samples. Alterations in multiple genes encoding co-signaling molecules, cytokines and chemokines that regulate immune responses were detected (Figure 3A). This included, for example, the exhaustion-related transcription factors *EOMES* and *TOX* [17,18], which were upregulated in CAR T cell products of non-responding patients compared to responding patients. Consistent with an effector T cell differentiation, we also observed increased expression of the *CD45RO, HLA-DRB1* and *CD86* genes. Among the upregulated genes in CAR T cell products of non-responding patients were also those previously reported to be expressed on senescent T cells [8,19], such as multiple killer lectin-like and killer cell immunoglobulin-like receptor genes, including *KLRG1*, *KLRD1* and *KLRC1*/2. Furthermore, CAR T cell products from non-responding patients were enriched in gene transcripts of *GZMB*, *GZMH*, *GZMA*, *CCL3*, *CCL4* and *CX3CR1*, which have been linked to an exhausted CD8+ T cell phenotype [20,21,22]. The apoptotic gene *FASLG,* previously described for senescent and exhausted T cells [23], was also upregulated in PD samples. In contrast, genes associated with T cell activation, including *IFNG*, *IL-15* and *CD69*, as well as genes associated with a less-differentiated T cell phenotype (*IL-7R*, *CD27*, *CCR7, Sell*) were down-regulated in CAR T cells from non-responding patients. The mRNA level of MYC, known to provide proliferative and pro-survival signals upon the engagement of T cell receptor (TCR) and IL-2 signaling [24], was reduced in CAR T cell products from non-responding patients. Of note, transcription factors *FOS* and *FOSB*, previously reported to be down-regulated in hyporesponsive T cells [25], were reduced in products from non-responding patients compared to products from responding patients.

We validated the Nanostring array data by quantitative real-time PCR (qPCR) of six selected genes using a larger cohort of CAR T cell products from patients who responded to the CAR T cell therapy (*n* = 9) in comparison to non-responding patients (*n* = 6). Consistently, the qPCR gene expression mirrored the findings in the array. Gene expression of *CCL4*, *GZMB*, *TOX*, *EOMES* and *CX3CR1* was significantly elevated, while *FOS* was down-regulated in CAR T samples from non-responding patients (Figure 3B). Furthermore, a co-expression pattern among the genes was detected. Thus, increased levels of *GZMB* positively correlated with increased levels of *CCL4* (R^2^ = 0.6026) and *CX3CR1* (R^2^ = 0.3681), and inversely correlated with *FOS* (R^2^ = 0.1592) in the CAR T samples. In addition, *CCL4* levels positively correlated with *CX3CR1* (R^2^ = 0.3909) and *EOMES* (R^2^ = 0.3088), while *CX3CR1* also positively correlated with *EOMES* (R^2^ = 0.2458) (Figure 3C). Altogether, our data revealed the transcriptomic signature in CAR T cell products, identified exhausted populations, and associated the transcriptomic profile with an exhausted phenotype of CAR T cells and reduced clinical response to treatment.

### 3.5. Reduced Ex Vivo Expansion of CAR T Cells Correlates with Exhausted Phenotype

**Figure 4:**
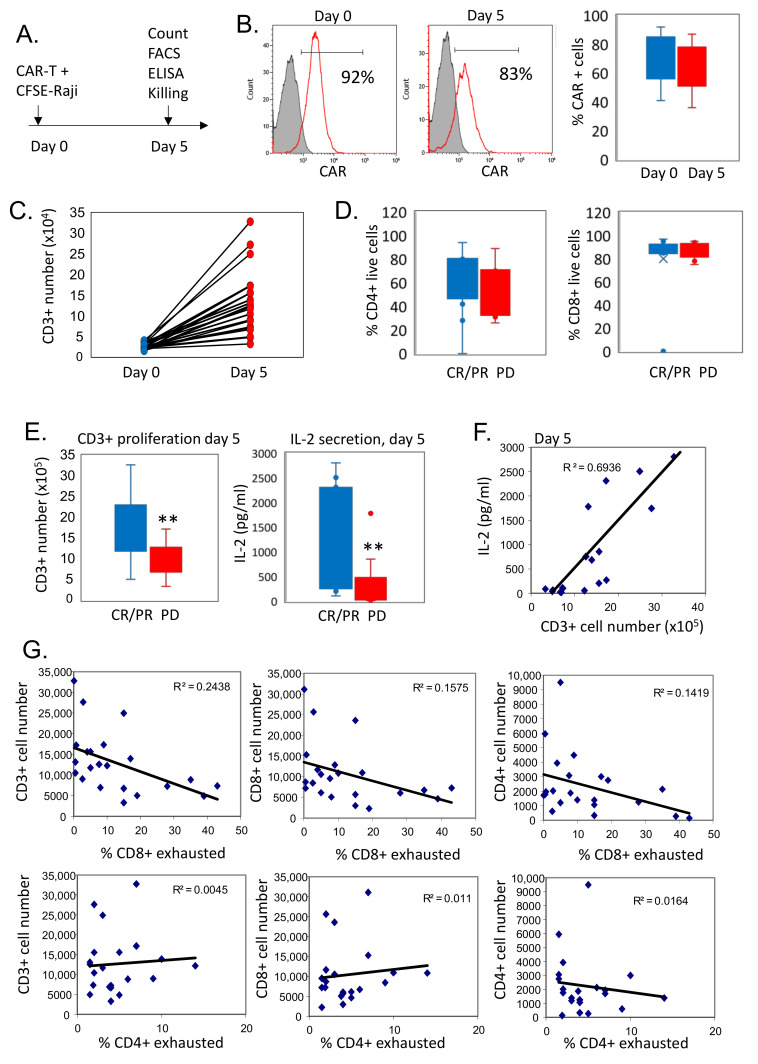
CAR T cell proliferative capacity and IL-2 secretion correlate with therapeutic response. (**A**) Schematic representation of co-culture experiment with CAR T cells and CFSE-labeled target Raji cells. (**B**) Representative histogram plots (left panel) and summarized data (right panel) showing levels of CAR expression in pre-infused CAR T cells before and after 5-day stimulation with CFSE-labeled CD19^+^ Raji cells (*n* = 22). Cells were pre-gated on CFSE-negative CD3^+^ T cells. (**C**) CAR T cells were co-cultured with CFSE-labeled Raji cells at a ratio of 1:1. Five days later, gated CFSE-negative CD3^+^ CD4^+^ or CD8^+^ CAR T cells were enumerated using 123count eBeads™ Counting Beads before co-culture and at day 5 of co-culture. (**D**) Percent of viable CD4^+^ and CD8^+^ CAR T cells was determined using 7-AAD staining and flow cytometry analysis at day 5 of co-culture with Raji cells. (**E**) Number of proliferated CD3^+^ cells (left panel) and IL-2 levels (pg/mL) in conditioned medium (right panel) at day 5 of co-culture, in each response group (CR/PR, *n* = 12; PD, *n* = 10); ** *p* < 0.01. (**F**) Spearman’s rho correlation (two-tailed) between the number of ex vivo proliferated CAR T cells and levels of IL-2 in the conditioned medium at day 5 of co-culture with target cells (*n* = 22). (**G**) Spearman’s rho correlation (two-tailed) between the number of ex vivo proliferated CD3^+^, CD4^+^, CD8^+^ CAR T cells at day 5 of co-culture with target cells, and frequency of exhausted (CD28^-^CD57^+^CD39^+^) CD8^+^ cells (upper panel) or exhausted CD4^+^ cells (lower panel) in manufactured CAR T products (*n* = 22).

The proliferative and survival capacities of CAR T lymphocytes strongly correlate with the antitumor activity of adoptively transferred T cells [6,26]. As standard release criteria, all CAR T cell products were able to lyse CD19^+^ cells and produce IFNγ in response to antigens [27]. To assess more stringent requirements that may functionally demonstrate the exhaustive phenotype shown in the CAR T cell products, we next examined the ex vivo expansion potential and IL-2 production of patient-derived CAR T cells (*n* = 22) upon exposure to tumor cells. CAR T cells were co-cultured with CD19-expressing CFSE-labeled Raji cells for five days (Figure 4A). To determine optimal expansion conditions, preliminary titration experiments were performed, evaluating the effect of a different effector: target (E:T) ratios. Equal numbers of CAR T cells and Raji targets cells (1:1 E:T ratio) in the starting culture were defined as optimal, inducing superior expansion and complete elimination of target cells at day 5 (Supplementary Figure S3).

Following the co-culture with CD19-expressing target cells, the CAR T cells underwent proliferation, preserving their CAR surface expression (Figure 4B). The CAR T cell samples demonstrated various levels of expansion, ranging from a 1.5-fold to 9.5-fold increase in the cell number observed on day 5 of co-culturing (Figure 4C). The proportion of CD4^+^ and CD8^+^ cells were similar in CAR T cells from responding (*n* = 12) and non-responding patients (*n* = 10) (Figure 4D). However, CAR T cells from patients who responded to therapy exhibited superior expansion and produced higher levels of IL-2 after a 5-day exposure to CD19 expressing tumor cells compared to CAR T cells obtained from patients not responding to CAR T cells (Figure 4E). Of note, IL-2 secretion correlated with the number of proliferating cells (R^2^ = 0.6936), therefore reflecting the degree of ex vivo CAR T proliferation and activation (Figure 4F). Importantly, we observed that ex vivo expansion potential of CAR T cells inversely correlated with the degree of CD8^+^ CAR T exhaustion. An increased percentage of exhausted CD8^+^ but not CD4+ CAR T cells in the manufactured product was associated with reduced cell proliferation following antigen exposure (Figure 4G). Altogether, our results suggest that an exhaustion phenotype is associated with diminished expansion capacity upon CAR stimulation and may be potentially linked with reduced in vivo efficacy of CAR T cells.

### 3.6. Antigen-Exposed CAR T Cells Preserve Cytotoxic Activity against CD19-Expressing Target Cells

**Figure 5:**
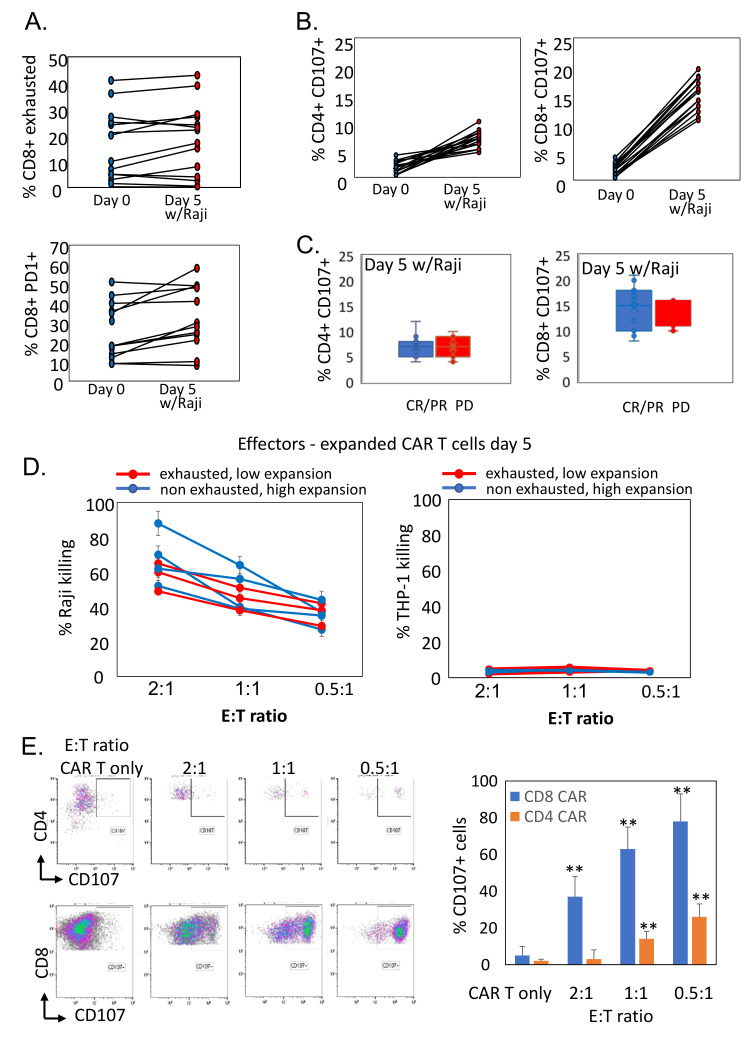
Antigen exposed CAR T cells preserve anti-tumor activity. (**A**) Frequency of exhausted and PD1-expressing CD8^+^ cells in paired samples of pre-infused CAR T cell products and expanded CAR T cells (*n* = 22) after 5 days of co-culture with Raji cells. (**B**) Frequency of degranulated CD107-expressing CD4^+^ and CD8^+^ cells in paired samples of pre-infused CAR T cell products and expanded CAR T cells (*n* = 22) after 5 days of co-culture with Raji cells; ** *p* < 0.01. (**C**) Percentages of degranulated CD107^+^ CD8^+^ and CD4^+^ T cells in antigen-exposed CAR T cells from patients in each response group (CR/PR, *n* = 12; PD, *n* = 10). (**D**) Percentage of specific lysis induced by antigen-exposed CAR-T cells against CFSE-labeled target tumor cells (Raji and THP-1) at indicated E:T ratios determined by FACS analysis at 4 h culture. Experiments were performed with three biological replicates. Data are presented as mean ± SD (** *p* < 0.01, two-sided Student’s *t*-test). CAR T cells with exhausted phenotype and low expansion rate (below 2.5-fold at day 5) are depicted in red (*n* = 4). CAR T cells with non-exhausted phenotype and high expansion rate (above 3-fold at day 5) are depicted in blue (*n* = 3). (**E**) Frequency of degranulation (CD107 expression) in expanded CD8^+^ and CD4^+^ CAR T cells that were repeatedly stimulated with CFSE-labeled Raji cells at indicated E:T ratios determined by FACS analysis at 4 h culture. Left panel—representative dop plots, right panel—summarized data presented as mean ± SD (** *p* < 0.01, two-sided Student’s *t*-test).

Next, we assessed whether antigen exposure promotes additional changes in CAR T cell phenotype. Notably, no change in the frequency of CD57^hi^CD39^hi^CD28^low^ CD8^+^ cells and PD1^+^CD8^+^ T cells was detected following 5-day expansion with CD19-expressing Raji cells (Figure 5A). However, CD107 expression was significantly upregulated on the cell surface of both CD4^+^ (2.1% ± 2% in non-expanded vs. 7.8% ± 3% in antigen-exposed, *p* < 0.0005) and CD8^+^ (3.5% ± 2% in non-expanded vs. 14.5% ± 5% in antigen-exposed, *p* < 0.0001) cells indicating the activation and degranulation of CAR T cells upon CAR engagement (Figure 5B). CAR T cells from both responding (CR/PR) and non-responding (PD) patients showed similar levels of degranulation (Figure 5C).

Lastly, to evaluate the function of CAR T cells, we examined their abilities to mediate anti-tumor cytotoxicity, after a 5-day expansion with CD19-expressing target cells. Following the expansion, CAR T cells (*n* = 7) were co-cultured at different E:T ratios with CFSE-labeled CD19-positive Raji lymphoma cells and CD19-negative THP1 myeloid leukemia cells for 4 h. Notably, antigen exposed CAR T cells promoted specific dose-dependent lysis of Raji cells, without detected cytotoxicity against THP-1 cells (Figure 5C). Low proliferative, exhausted CAR T cells remained functional, demonstrating specific cytotoxic activities compared with those of non-exhausted highly proliferative cells (Figure 5D). Repetitive tumor challenge with Raji cells induced profound CAR T degranulation, as demonstrated by increased levels of CD107 expression in both CD4^+^ and CD8^+^ cells. Interestingly, a lower dose of target cells in the co-culture promoted higher induction of CD107 (Figure 5E). Collectively, these results indicate that CAR T cells sustain their in vitro effector function following CAR-mediated ex vivo expansion, despite the increased exhaustion phenotype and reduced proliferative capacity.

## 4. Discussion

T cell exhaustion has recently been identified as an important correlate of clinical response in patients undergoing CAR T therapy [28,29], highlighting the need for better immune profiling of T cell dysfunctionality as a relevant biomarker for immune therapy trials. Therefore, the goal of the current work was to identify the phenotypic, molecular and functional signatures associated with CAR T cell exhaustion and reduced clinical response in patients with B cell malignancies. Both phenotypic and transcriptional studies revealed that CAR T cells with a mixed exhausted and senescent phenotype are increased in the manufactured products of non-responding patients. Interestingly, CD8^+^ cells in CAR T products of responding patients had a less differentiated phenotype, with a reduced proportion of EM (CD45RA^-^CD62L^-^) cells, compared to the CD8+ counterparts in non-responding patients. Of note, a significantly lower content of EM CD8^+^ cells was also observed in PB samples of responding patients prior to therapy. These results were confirmed by transcriptomic analysis, showing increased *CCR7* and reduced *CD45RO* gene expression in CAR T samples from CR patients in comparison to CAR T cells from patients with PD. These data are in agreement with previous studies that linked T cell exhaustion with a differentiated phenotype [6,30]. Furthermore, recent work shows that IL-9-polarized CAR T cells leading to reduced exhaustion and increased anti-tumor activity, demonstrate less differentiated phenotype with reduced frequency of EM cells [31].

Recent studies have shown that inhibitory receptors (i.e., PD-1, CTLA-4, LAG3) that are expressed on exhausted T cells can also be expressed by functional effector T cells and may be present on memory T cell populations and therefore lack specificity [32,33,34]. Therefore, our exhaustion phenotypic profiling was not based solely on the recognition of inhibitory molecules but rather we used a combination of reduced CD28 together with up-regulated CD39 and CD57 for the recognition of the exhausted CAR T cell population. Indeed, the frequency of exhausted CD8^+^ CAR T cells in the CAR T cell products correlated with PD-1 and LAG-3 cell surface expression levels, hence supporting their applicability and relevance to the impaired T cell phenotype. Furthermore, the increased percentage of exhausted CD8^+^ CAR T cells inversely correlated with CD127 expression levels. It is well established that CD8^+^ memory cells persist longer in the absence of antigen stimulation via IL-7- and IL-15-driven signals, requiring co-expression of both CD122 (also known as IL-2Rβ) and CD127, which are the key receptor subunits for responsiveness to IL-7 and IL-15 [35,36]. Thus, our results might indicate that CAR T cells with low CD127 expression lack the responsiveness to IL-7 and IL-15, and may persist poorly following adoptive transfer to the patient.

CD57 was originally recognized as a differentiation antigen on the surface of NK cells [37] and subsequently associated with T cell dysfunction, senescence and exhaustion [38,39]. CD39 is a surface-expressed ATP ectonucleotidase, and its expression is associated with a specific subset of CD8^+^ T cells with exhaustion features in several cancer models [40,41,42]. Stimulation of PBMCs from breast cancer patients with anti-CD3/anti-CD28 was sufficient to trigger CD39 expression [40]. These results may explain the increase in CD39-positive exhausted CD8^+^ population upon the CAR T manufacturing process. It is conceivable that T cell activation required for efficient transduction and expansion of CAR T cells may promote the acquisition of an exhausted phenotype in a subset of patients, therefore predisposing insufficient clinical responses.

Our observations were further supported by transcriptomic studies, indicating dysregulation of multiple genes associated with T cell exhaustion and senescence, distinct cytokine expression profile, and decreased expression of co-stimulatory molecules in CAR T cells from non-responding patients compared with samples from CAR T patients achieving CR. Thus, it appears that increased expression of genes encoding for granzymes, *GZMA*, *GZMB* and *GZMH*, elevated levels of *CCL3*, *CCL4*, increased levels of transcription factors *TOX* and *EOMES*, reduced expression of *FOS* and *FOSB,* and reduced levels of genes associated with naive T cell phenotype (*IL-7R*, *CD27*, *CCR7, Sell*) compose the molecular signature of exhausted CAR T from non-responding patients. These results aligned with data from a previous study, which demonstrated that terminally exhausted CD8^+^ tumor-infiltrating lymphocytes (TILs) in a mouse melanoma model displayed a similar molecular signature, expressing high levels of *Gzma*, *Gzmb*, *Klrg1, CXC3CR1, Ccl3* and *Ccl4* [21]. A similar phenotype was observed in dysfunctional CD8^+^ T cells in patients with acute myelogenous leukemia (AML) displaying co-existence of exhaustion and senescence signature characterized by reduced CD28 and CD127, high CD57 expression, and high PD-1 [43]. Our data are also consistent with those of Wherry et al. showing that FOS and FOSB transcription factors are decreased in exhausted T cells during chronic viral infection [25].

The currently observed phenotypic and transcriptomic findings were further supported by functional studies showing that high frequency of CAR T cells with exhausted phenotype in the cellular product was associated with reduced proliferation and decreased IL-2 production in response to CAR T stimulation. The latter results are consistent with previous studies demonstrating reduced IL-2 secretion and reduced proliferation of exhausted T cells [25]. The loss of distinct T cell functions during chronic viral infection occurs in a hierarchical manner, while IL-2 production and robust proliferation are the first functions to be lost [44]. In addition, post-transcriptional regulation of cytokine production may dampen functional responses of exhausted CAR T cells upon sustained TCR or CAR stimulation. Indeed, it was recently shown that post-transcriptional events mediate CD8^+^ T cell dysfunction in tumor-infiltrating lymphocytes (TILs) [45]. Therefore, loss of *IFNγ* mRNA stability that can be promoted due to lack of CD28 co-stimulation [45], or translational block in IFNγ production that can be mediated by PD-1 signaling in exhausted cells [46], may further diminish CAR T cell effectiveness.

Nevertheless, our data indicate that exhausted CAR T cells are not inert. Of note, prolonged antigen-exposed CAR T cells with an exhausted phenotype demonstrated killing of CD19^+^ targets in vitro, comparable with non-exhausted CAR cells. Similarly, CAR T cell degranulation was triggered in vitro after target recognition regardless of the cell phenotype. These data suggest that exhausted CAR T cells undergo insufficient proliferation and IL-2 production while maintaining their cytolytic activity against NHL cells. Several lines of evidence suggest that exhausted or senescent T cells preserve anti-tumor activity. It was shown that CD57^+^PD1^+^ PB T cells from the patients with non-small cell lung cancer exhibited cytotoxic potencies but impaired proliferative capability [47]. Accordingly, terminally exhausted TILs in melanoma patients demonstrated reduced proliferative capacity, but profound anti-tumor cytotoxic activity, probably due to elevated levels of granzyme B [21]. Altogether, our results suggest that impaired proliferative capacity of exhausted/senescent CAR T cells may be translated into diminished in vivo expansion and therefore reduced effectiveness in non-responding patients. Recent studies suggest that long-term persistence of the infused CAR T cells is crucial for sustained response. Moreover, Melenhorst and colleagues recently reported in a seminal paper a highly activated CD4^+^ population dominating the CAR T cell population at the later time points in two CLL patients with a decade-long remission post CAR T treatment [48]. These data speak for the importance of CD4^+^ CAR T counterpart for long-term cytotoxicity against CD19-expressing cells and support our findings suggesting that CD4^+^ CAR T cells may be less prone to exhaustion compared with CD8^+^ cells.

The mechanism of CAR T cell exhaustion remains poorly understood and may be affected by the donor properties, the scFv [49,50], the co-stimulatory domain [49,51] or other features. Murine models demonstrated a higher risk of CD8 CAR T cell exhaustion following TCR-mediated ligand binding of CD8 but not CD4 cells [52]. These results further support our current findings, indicating that CD4^+^ CAR T cells are less prone to exhaustion upon excessive activation.

The concept that CAR T exhaustion may be prevented or even reversed is gaining attention, providing a potential solution to the lack of adequate clinical responses in B cell malignancies. Upregulation of inhibitory receptors such as PD-1 in response to antigen exposure may significantly inhibit CAR T cells activity. PD-1 blockade was suggested to be a causative mechanism of exhaustion reversion in exhausted progenitor TILs and anti-tumor activity in melanoma patients receiving anti-PD-1 therapy [21,53]. Moreover, strategies for interference of the PD-1 axis that were developed recently emerge as a promising approach for reinvigorating CAR T cells in clinical settings [54,55] but failed to improve TCR-based adoptive cell therapies [56]. Progressive T cell exhaustion is established and maintained by the action of the transcription factor and epigenetic modifier TOX [18,57], suggesting that its inhibition in CAR-T cells may promote the effector pathway with its attendant higher cytokine levels and cell killing. Accordingly, the murine model has shown that CAR T deficient for *tox1* and *tox2* are more effective than wild-type CARs in suppressing tumor growth [57]. Additionally, overexpression of c-JUN, an AP1 family transcription factor involved in T cell activation, was able to reverse the dysfunctional state of exhaustion-prone CAR T cells, resulting in improved function and reduced expression of PD-1 and CD39 [58]. Another intriguing approach to prevent or reverse the CAR T cell exhaustion relies on the suppression of CAR-mediated signaling, therefore preventing tonic signaling in CAR T cells. Notably, the tyrosine kinase inhibitor dasatinib inhibits CAR signaling during manufacturing, to an extent sufficient to reduce exhaustion from tonic signaling and enhance CAR T cell efficacy in vivo [59]. Finally, our current understanding of T cell fitness and function should be translated into specifically tailored clinical decisions in order to improve CAR T cell treatment efficacy. Previous studies suggest that multiple myeloma (MM) patients that underwent leukapheresis early after diagnosis had greater expansion in the CAR T cell product than those that underwent leukapheresis later in the disease course [60]. Therefore, it is conceivable that introducing CAR T cell therapy earlier in a patient’s treatment paradigm will increase CAR T expansion and thus improve treatment efficacy. Alternatively, patients with increased T cell exhaustion in the leukapheresis product may benefit from novel manufacturing strategies, such as enrichment protocols for less differentiated T cell subsets eliminating dysfunctional cells.

## 5. Conclusions

Our results reveal potential cellular and molecular markers of CAR T cells resistance, identifying an enrichment of differentiated exhausted/senescent CD8^+^ T cells as the main feature observed in PB and CAR T products of non-responding patients. Further delineating these mechanisms will guide future T cells engineering studies aiming to enhance the efficacy and response durability of CAR T therapy in B cell malignancies.

## Figures and Tables

**Table 1:** Baseline characteristics of the patients treated with CD19 CAR T cells.

Characteristic	Value
Median age, years (range)	48.01 (27–73)
**Gender**	
Male, *n* (%)	24 (57%)
Female, *n* (%)	18 (43%)
**Diseases**	
DLBCL *n*, (%)	24 (57%)
Mantle *n*, (%)	3 (7%)
Follicular *n*, (%)	5 (12%)
ALL *n*, (%)	5 (12%)
CLL *n*, (%)	5 (12%)
**Response**	
CR *n*, (%)	24 (57%)
PR *n*, (%)	4 (10%)
PD *n*, (%)	14 (33%)
**Toxicity (CRS)**	
No *n*, (%)	5 (12%)
Grade 1–2 *n*, (%)	32 (76)
Grade 3–4 *n*, (%)	5 (12%)
**Neurotoxicity**	
No *n*, (%)	22 (53%)
Grade 1–2 *n*, (%)	12 (27%)
Grade 3–4 *n*, (%)	8 (20%)

Abbreviations: DLBCL, diffuse large B-cell lymphoma; CRS, cytokine release syndrome; ALL, acute lymphoblastic leukemia; CLL, chronic lymphocytic leukemia; CR, complete response; PR, partial response; PD, progressive disease.

## Data Availability

Not applicable.

## Supplementary Materials

The following supporting information can be downloaded at: https://www.mdpi.com/article/10.3390/cells11071140/s1, Figure S1: Gating strategy of exhausted T cells; Figure S2: Chemokine receptor expression and Treg frequency in CAR T and peripheral blood T cells; Figure S3: Optimization of CAR T cell expansion upon exposure to Raji cells; Table S1: Primers sequences; Table S2: Clinical characteristics and toxicities of CAR T cells according to objective response; Table S3: Flow cytometry antibodies
