# Supplementary material for: Molecular and Functional Signatures Associated with CAR T Cell Exhaustion and Impaired Clinical Response in Patients with B Cell Malignancies
Source: Cells. 2022 Mar 28;11(7):1140. doi: 10.3390/cells11071140 (PMC8997745; doi:10.3390/cells11071140)
Supplement: Supplementary file 1 [file cells-11-01140-s001.zip › cells-1618888-supplementary.pdf]

# Supplementary Materials

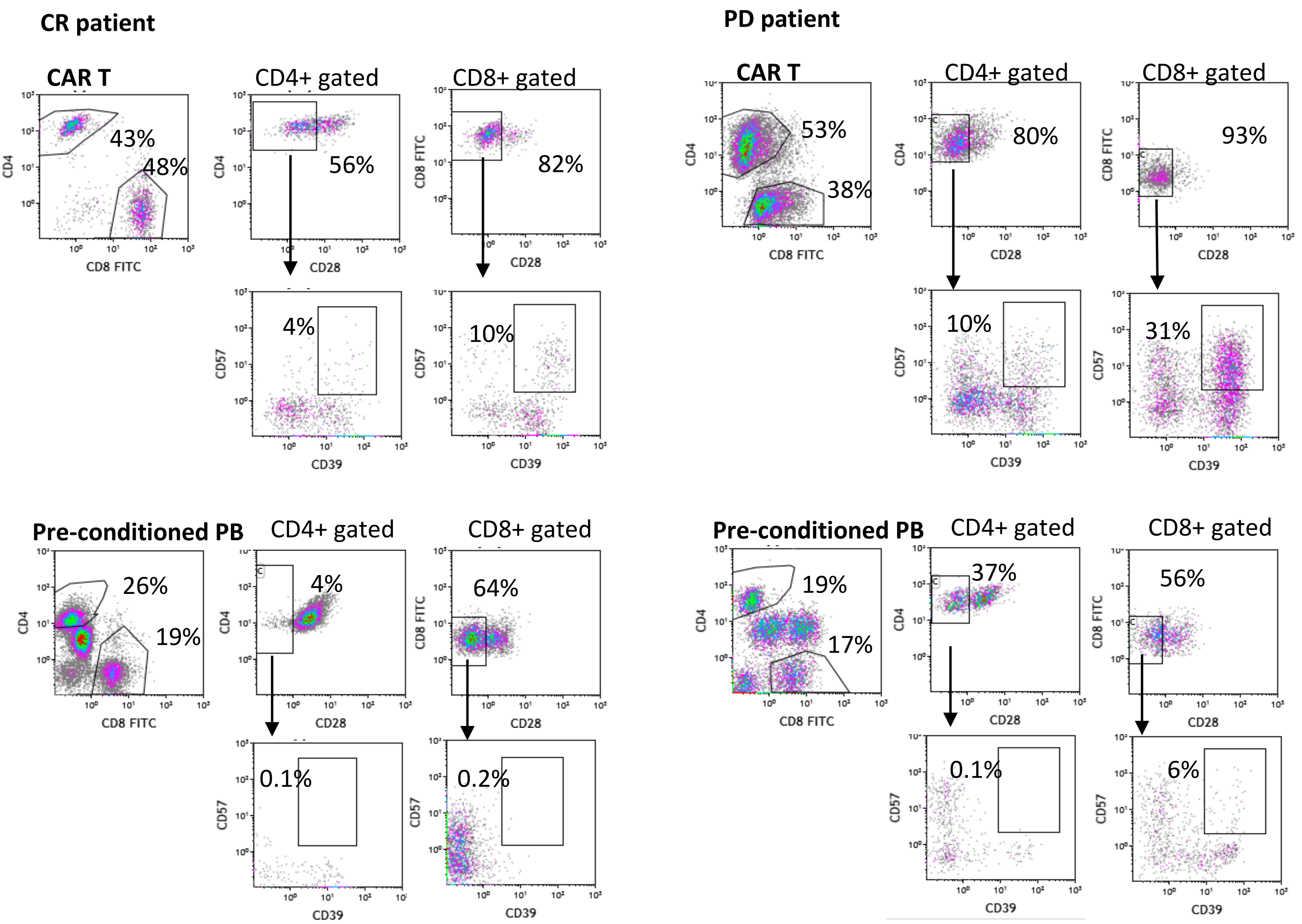


**Figure S1. Gating strategy of exhausted T cells.** Pre-infused CAR T cell products and PB samples at the time of leukapheresis were analyzed by multiparameter flow cytometry. Representative flow cytometry dot plots examining the frequency of exhausted population (CD28-CD57+CD39+) in CD8+ and CD4+ T cells.

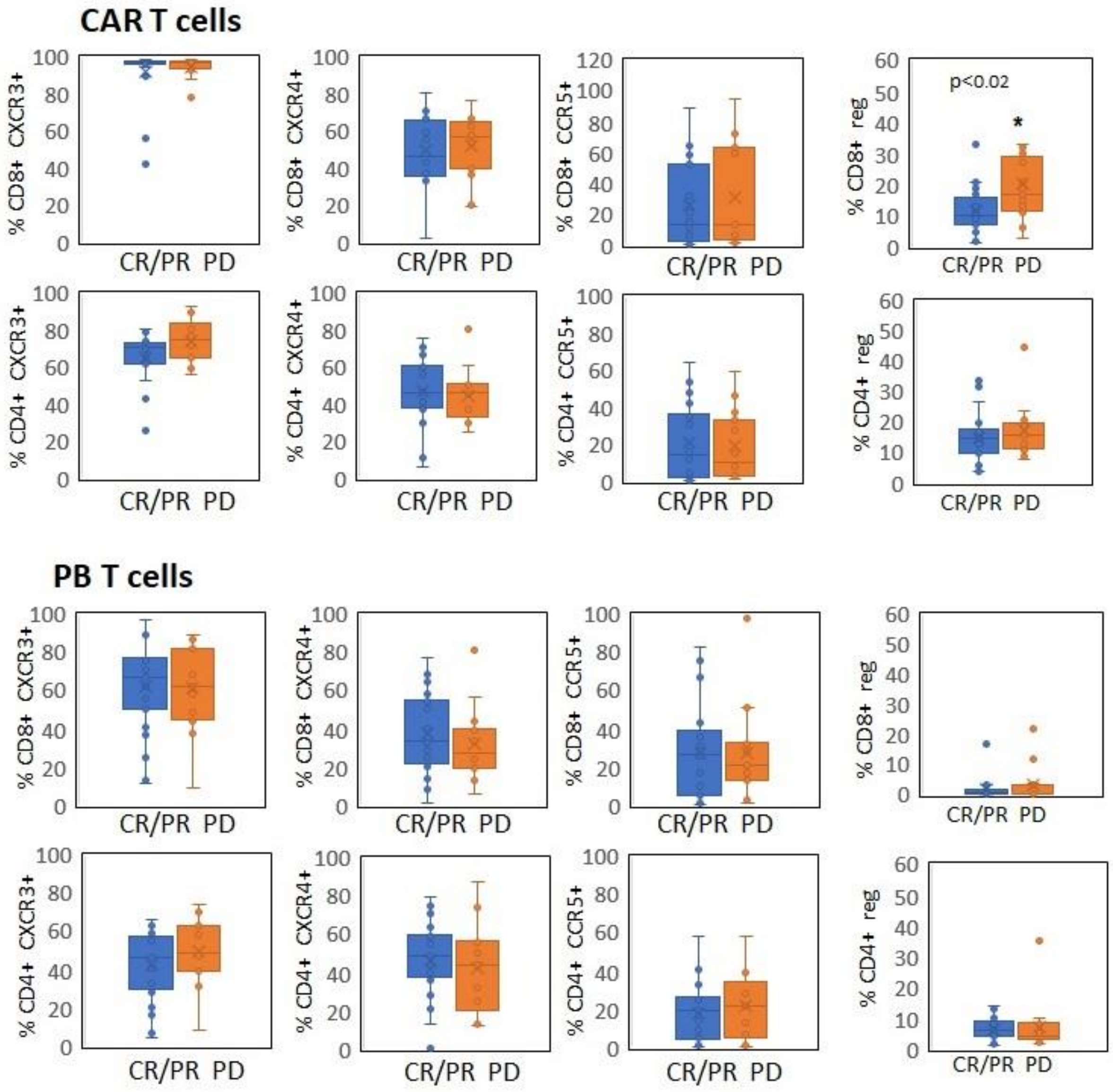


**Figure S2. Chemokine receptor expression and Treg frequency in CAR T and peripheral blood T cells.**

Pre-infused CAR T cell products and PB samples at the time of leukapheresis from patients in each response group (CR/PR, n= 28; PD, n=14) were analyzed by multiparameter flow cytometry. Frequency of CXCR3+, CXCR4+, CCR5+ and regulatory (CD25+CD127-low) CD8+ and CD4+ cells in paired samples of PB and manufactured CAR T cells from CR/PR and PD patients.

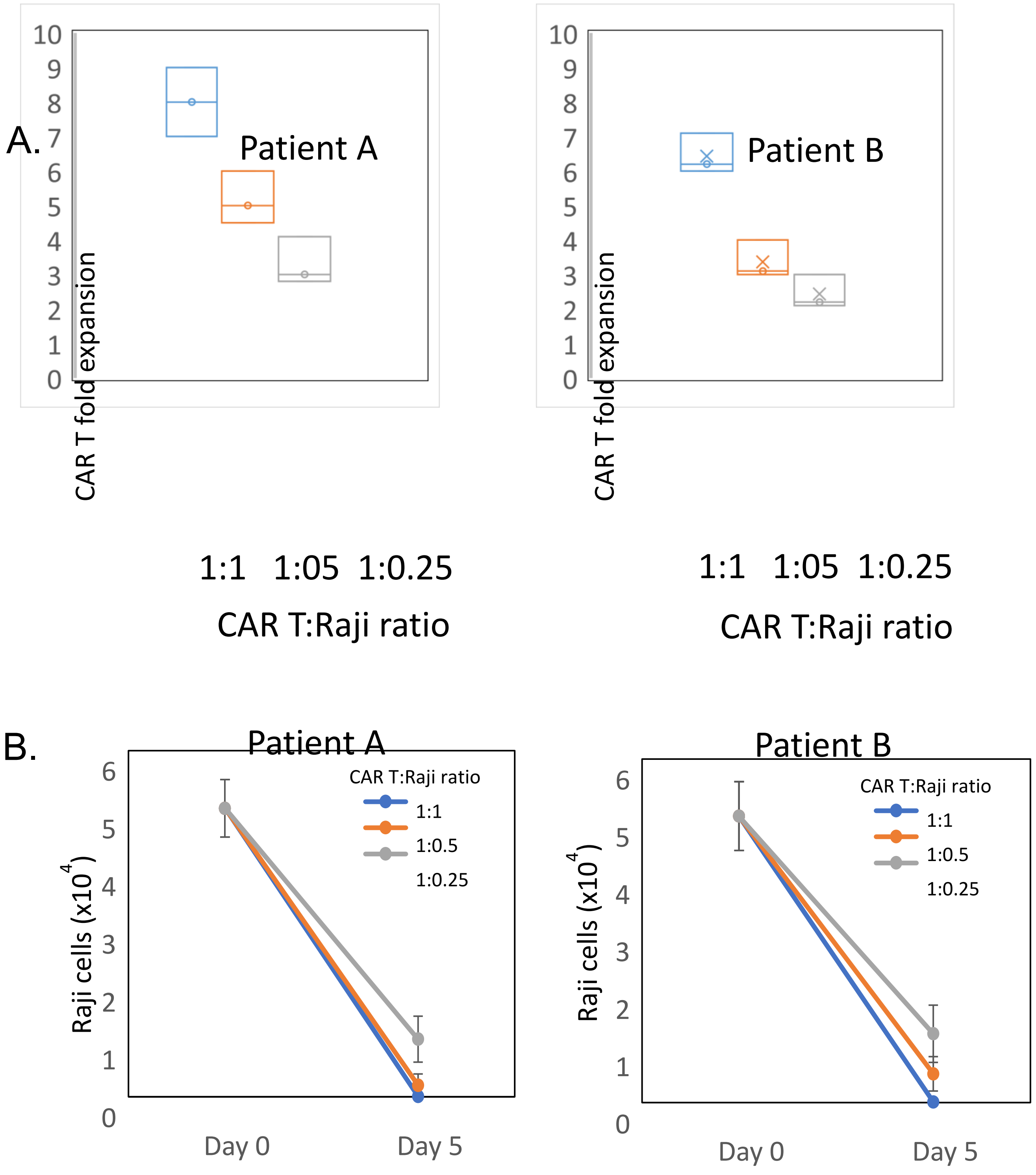


**Figure S3. Optimization of CAR T cell expansion upon exposure to Raji cells.** CAR T cells were co-cultured with CFSE-labeled Raji cells at ratios of 1:1, 1:0.5,and 1:0.25. Five days later, gated CFSE-negative CD3+ CD4+ or CD8+ CAR T cells were enumerated using 123count eBeads™ Counting Beads before co-culture and at day 5 of co-culture. CAR T fold expansion was calculated (A). The number of viable (PI-negative) CFSE-labeled Raji tumor cells at day 0 and following 5 days of co-culture with CAR T cells at ratios of 1:1, 1:0.5,and 1:0.25 (B).

**Table S1**: Primers sequences

| gene | forward | reverse |
|---|---|---|
| FOS | AGGAGGGAGCTGACTGATACACT | TTTCCTTCTCCTTCAGCAGGTT |
| TOX | CCTGCCTGGACCCCTACTAT | CTGGCTGGCACATAGTCCTG |
| EOMES | AGGCGCAAATAACAACAACACC | ATTCAAGTCCTCCACGCCATC |
| CX3CR1 | GTGGTGCTGACAAAGCTTGGAA | TCACTGGGTGCCATCGTAAGAA |
| CCL4 | GCTTTTCTTACACTGCGAGGA | CCAGGATTCACTGGGATCAG |
| GZMB | GTGCAAGGAAGATCGAAAGTGC | GCATGCCATTGTTTCGTCCA |

**Table S2. Clinical characteristics and toxicities of CAR T cells according to objective response.** Abbreviations; DLBCL, diffuse large B-cell lymphoma; CRS, cytokine release syndrome; ALL, acute lymphoblastic leukemia; CLL, chronic lymphocytic leukemia; CR, complete response; PR, partial response; PD, progressive disease.

| **Characteristic** | **CR/PR** n=28 | **PD** n=14 |
|---|---|---|
| Median age, years | 46.1 | 53.3 |
| Gender | | |
| Male, n (%) | 19 (68%) | 5 (36%) |
| Female, n (%) | 9 (32%) | 9 (64%) |
| Disease | | |
| DLBCL, n (%) | 14 (50%) | 10 (71%) |
| Mantle, n (%) | 2 (7%) | 1 (7%) |
| Follicular, n (%) | 5 (18%) | 0 |
| ALL, n (%) | 5 (18%) | 0 |
| CLL, n (%) | 2 (7%) | 3 (21%) |
| Toxicity | | |
| CRS | | |
| no, n (%) | 4 (14%) | 1 (7%) |
| Grade 1-2, n (%) | 22 (79%) | 10 (71%) |
| Grade 3-4, n (%) | 2 (7%) | 3 (21%) |
| Neurotoxicity | | |
| no, n (%) | 14 (50%) | 8 (57%) |
| Grade 1-2, n (%) | 7 (25%) | 5 (36%) |
| Grade 3-4, n (%) | 7 (25%) | 1 (7%) |

**Table S3**: Flow cytometry antibodies

| Antibody | Fluorochrome | Clone | Provider | Dilution |
|---|---|---|---|---|
| CD4 | FITC | OKT4 | Tonbo | 1:100 |
| CD25 | PE | BC96 | Tonbo | 1:100 |
| CD3 | APC | UCHT1 | Biogems | 1:100 |
| CD8 | PE-Cy7 | OKT8 | Biogems | 1:100 |
| CD45RA | PercP-Cy5.5 | HI100 | Biogems | 1:100 |
| CD45RO | PercP-Cy5.5 | UCHL1 | Biogems | 1:100 |
| CD127 | APC | A019D5 | Biolegend | 1:100 |
| CD62L | PE | DREG-56 | Biolegend | 1:100 |
| CD57 | APC | HCD57 | Biolegend | 1:100 |
| CD39 | PE | A1 | Biolegend | 1:100 |
| CD107 | PercP-Cy5.5 | H4A3 | Biolegend | 1:100 |
| PD1 | PE | EH12.2H7 | Biolegend | 1:50 |
| LAG3 | PercP-Cy5.5 | 11C3C65 | Biolegend | 1:50 |
| CCR5 | PE | J418F1 | Biolegend | 1:100 |
| CCR6 | APC | G034E3 | Biolegend | 1:50 |
| CCR7 | APC | G043H7 | Biolegend | 1:100 |
| CXCR3 | PE | G025H7 | Biolegend | 1:100 |
| CXCR4 | APC | 12G5 | Biolegend | 1:100 |
